# Mangelernährung bei geriatrischen Patient*innen: Risikofaktor stationäre Langzeitpflege?

**DOI:** 10.1007/s16024-021-00353-z

**Published:** 2021-06-11

**Authors:** Fabian Graeb, Reinhold Wolke

**Affiliations:** Fakultät Soziale Arbeit, Bildung und Pflege, Institut für Gesundheits- und Pflegewissenschaften, Hochschule Esslingen – University of Applied Sciences, Flandernstraße 101, 73732 Esslingen am Neckar, Deutschland

**Keywords:** MUST, Geriatrie, NutritionDay, Krankenhaus, Screening, MUST, Geriatrics, NutritionDay, Hospital, Screening

## Abstract

**Hintergrund:**

Mangelernährung ist ein nach wie vor herausforderndes Problem in der Krankenhausversorgung, speziell bei geriatrischen Patient*innen. Dennoch findet das Thema in der Praxis nur wenig Beachtung.

**Ziel und Methodik:**

Im Zentrum der vorliegenden Datenanalyse steht die Fragestellung, inwiefern sich der Ernährungsstatus von zuhause lebenden geriatrischen Patient*innen, von in der stationären Pflege lebenden, unterscheidet. Hierfür wurden Daten aus insgesamt 4 Erhebungen (3-mal nutritionDay plus eine zusätzliche Erhebung) zusammengefasst. Es konnten 258 Patient*innen (≥ 65 Jahre) in die Auswertung aufgenommen werden; ein Mangelernährungsrisiko wurde anhand des Malnutrition Universal Screening Tool (MUST), eine manifeste Mangelernährung anhand der ESPEN-Kriterien festgestellt.

**Ergebnisse:**

Zu Hause leben 86,0 % (*n* = 222) der Patient*innen, in stationären Pflegeeinrichtungen 14,0 % (*n* = 36). Die in der stationären Pflege lebenden Patient*innen weisen eine ausgeprägtere Morbidität auf, sichtbar anhand der größeren Anzahl an in der Klinik verbrachten Nächten in den letzten 12 Monaten (Mdn 10,0 vs. 5; *p* 0,007), der höheren Anzahl der Medikamente (Mdn 9,0 vs. 7,0; *p* 0,002) sowie stärkeren Einschränkungen beim Gehen (Mdn 3,0 vs. 1,0; *p* < 0,001). Sie sind signifikant älter (Mdn 86,0 vs. 78,0 Jahre; *p* < 0,001) und weisen einen tendenziell höheren Anteil manifester Mangelernährung auf (35,7 %; *n* = 10 vs. 20,1 %; *n* = 40; *p* 0,062).

**Schlussfolgerung:**

Sowohl zu Hause als auch in der stationären Langzeitpflege lebende geriatrische Patient*innen weisen einen erheblichen Anteil an Mangelernährung auf. Ein zuverlässig durchgeführtes Screening zu Beginn und im Verlauf des Klinikaufenthaltes ist in jedem Fall dringend erforderlich, da nur so die Betroffenen erkannt werden. Ein regelmäßiges Screening im ambulanten Bereich wie auch der stationären Langzeitpflege ist ebenso erforderlich.

## Abstract (en)

**Background:**

Malnutrition remains a challenging problem in hospitals, especially among geriatric patients. The background and causes are actually well known but still underestimated in clinical practice.

**Aim and methods:**

This study evaluated possible differences in rates of malnutrition risk and malnutrition in geriatric patients living at home and geriatric patients living in residential nursing homes. For this purpose, data from a total of 4 surveys (3 times nutritionDay plus 1 additional survey) were combined and evaluated. In this secondary analysis, 258 patients 65 years and older are included. Malnutrition risk is determined by the malnutrition universal screening tool (MUST) and malnutrition by the ESPEN criteria.

**Results:**

Of the patients 86.0% (*n* = 222) live at home and 14.0% (*n* = 36) in residential nursing homes. The patients living in nursing homes have a higher morbidity and need of care. Indicators for the increased morbidity are a larger number of nights spent in the clinic in the last 12 months (mdn 10.0 vs. 5; *p* 0.007), higher number of daily drug intake (mdn 9.0 vs. 7.0; *p* 0.002) and greater limitations in walking abilities (mdn 3.0 vs. 1.0; *p* < 0.001). They are significant older (median 86.0 vs. 78.0; *p* < 0.001) and show a higher percentage of manifest malnutrition (35.7%; *n* = 10 vs. 20.1%; *n* = 40; *p* 0.062).

**Conclusion:**

Geriatric patients living at home and in residential nursing homes show high rates of malnutrition. A malnutrition screening at the beginning and during hospital stay is urgently required in every case as this is the only way to identify those affected. In addition, regular screening in outpatient departments as well as in nursing homes is also necessary.

## Hintergrund

Mangelernährung stellt speziell unter geriatrischen Patient*innen ein weit verbreitetes Phänomen dar. So weisen etwa laut Screeninginstrument Mini Nutritional Assessment (MNA) 17–30 % der geriatrischen Patient*innen eine Mangelernährung und weitere 38–65 % ein Mangelernährungsrisiko auf (Kiesswetter et al. 2016). Die Anteile variieren damit erheblich, u. a. abhängig von Klinikfachabteilung und genauer Zusammensetzung der Gruppe, etwa hinsichtlich der Pflegebedürftigkeit. Verschiedene Studien und Reviews haben sich mit den Ursachen einer Mangelernährung im Alter befasst. So beschreiben etwa Volkert et al. (2019a) die möglichen Ursachen einer Mangelernährung im Alter anhand des reviewbasierten Modells Determinants of Malnutrition in Aged Persons (DoMAP). Darin werden eine *geringe Nahrungszufuhr*, ein *erhöhter Bedarf* und eine *reduzierte Bioverfügbarkeit* als zentrale Entstehungsmechanismen einer Mangelernährung benannt. Auf diese wirken Faktoren ein, die diese direkt verursachen, wie beispielsweise *Erbrechen, Dysphagie, Vergessen zu essen, Entzündungen* oder eine *erhöhte Stoffwechselrate*. Weitere Determinanten wirken eher indirekt, wie etwa eine *Demenz*. Hierdurch vergessen die Betreffenden zu essen, was wiederum zu einer verringerten Zufuhr und in der Folge einer Mangelernährung führt. Weitere Beispiele dieser indirekt wirkenden Faktoren sind *M. Parkinson, Apoplex, Schmerzen, Operationen, COPD* oder *Medikamente*. Hinzu kommen alterstypische Veränderungen, die das Mangelernährungsrisiko allgemein und über verschiedene Mechanismen erhöhen, wie etwa *Polypharmazie, Multimorbidität* oder *Gebrechlichkeit* (Volkert et al. 2019a). Dieses elaborierte Modell verdeutlicht, dass das Phänomen Mangelernährung im Alter eigentlich gut erforscht ist. Dennoch zeigt sich immer wieder, dass das Thema in der klinischen Praxis kaum Beachtung findet. So wird in deutschen Krankenhäusern eine Mangelernährung immer noch zu selten erkannt, was wiederum dazu führt, dass selbst bei schwer mangelernährten Patient*innen nur selten eine gezielte Therapie erfolgt (Volkert et al. 2020). Der jährlich stattfindende nutritionDay hat aufgezeigt, dass dieses Problem keine Besonderheit des deutschen Krankenhauswesens ist. In einer norwegischen Auswertung von nutritionDay-Daten zeigt sich demnach ein ähnliches Bild mit einer hoher Prävalenz an Mangelernährung (30 %) bei gleichzeitig selten stattfindenden Ernährungsinterventionen. Nur 41 % der Betroffenen erhalten demnach eine entsprechende Therapie, während auch 22 % der gut ernährten Patient*innen eine Therapie erhalten (Henriksen et al. 2017). Damit kommt es zu selten zu Interventionen, und diese erfolgen offenbar eher ungezielt. Die Folgen dieses oftmals nichtbehandelten Problems sind jedoch erheblich. So ist ein Mangelernährungsrisiko u. a. mit einem erhöhten Sturzrisiko (Eglseer et al. 2020), der verringerten Chance, nach Hause entlassen zu werden, und einer erhöhten Mortalität assoziiert (Cardenas et al. 2020).

Die vielfach beschriebenen und weit verbreiteten Defizite im Ernährungsmanagement führten schließlich zur Entwicklung des Forschungsprojektes „*Prävention und Behandlung von Mangelernährung bei geriatrischen Patienten im Krankenhaus*“. Dieses wurde vom Bundesministerium für Bildung und Forschung (BMBF) im Rahmen des *Programms Soziale Innovationen für Lebensqualität im Alter (SILQUA-FH)* gefördert (01/2017–06/2020; Förderkennzeichen 13FH011SX6) (Wientjens et al. 2018).

Im Zuge des Projektverlaufs wurden verschiedene Datenerhebungen durchgeführt. Es zeigte sich dabei, dass auch geriatrische Patient*innen als Zielgruppe des Projektes keine homogene Gruppe darstellen, sondern sich diese Patientengruppe in vielen Aspekten sehr heterogen zusammensetzt. So stellen sich wesentliche gesundheitliche Faktoren, wie Morbidität oder Pflegebedürftigkeit, sehr unterschiedlich dar. Jeder Praktiker kennt vermutlich einerseits den selbstständig zu Hause lebenden 90-jährigen Senior, bei gutem Gesundheitszustand, wie auch den schwerstpflegebedürftigen und multimorbiden 70-jährigen Pflegeheimbewohner. Die individuelle Wohnsituation, ob selbstständig zu Hause oder in einer Pflegeeinrichtung lebend, stellt eine mögliche Differenzierungsmöglichkeit dar. In den gesichteten Studien wurde bislang stets nur zwischen Settings unterschieden, also etwa im Vergleich ambulante Pflege, stationäre Langzeitpflege und Krankenhaus. Es wurde aber nicht untersucht, ob und inwiefern sich die Ernährungsstatus von älteren Patient*innen in der Klinik unterscheiden, wenn diese in der stationären Langzeitpflege oder zu Hause leben. Diese Forschungslücke soll hier geschlossen werden. Anhand der im Projekt gesammelten Daten sollte daher in der vorliegenden Arbeit ermittelt werden, inwiefern sich der Ernährungsstatus von geriatrischen Patient*innen, die zu Hause leben, von denen, die in der stationären Pflege leben, unterscheidet.

## Methodik

Beide in das Projekt involvierte Kliniken haben mit ihren Projektstationen an den nutritionDays 2017, 2018 und 2019 teilgenommen. Der nutritionDay findet seit 2006 an einem Stichtag im November statt. An diesem werden in Kliniken weltweit Daten zu den dann stationär aufgenommenen Patient*innen erhoben, hinsichtlich deren Ernährungsstatus, aktuellem Ernährungsverhalten, Therapie und dem durchgeführten Ernährungsmanagement sowie dem klinischen Outcome 4 Wochen später (Schindler et al. 2017). In die vorliegende Auswertung flossen die Ergebnisse der standardisierten nutritionDay-Fragebogen 2a/b (allgemeine medizinische Informationen zu den Patient*innen aus der Akte), 3a/b (Befragung der Patient*innen/Betreuer*innen zu Ernährungsstatus, Mobilität, subjektiver Gesundheit und Ernährung vor Krankenhausaufenthalt) sowie die Outcome-Parameter Dauer des Klinikaufenthalts, Entlassart und Versterben ein (Hiesmayr 2020). Diese Bogen sind auf den Seiten des nutritionDay-Projektes online einsehbar (https://www.nutritionday.org/de/).

Ferner sollten anhand einer weiteren Erhebung in 2019 Veränderungen des Ernährungsstatus bei geriatrischen Patient*innen im Zuge des Krankenhausaufenthaltes erfasst werden. Hierbei wurden ebenfalls die nutritionDay-Instrumente 2a/b, 3a/b und Outcome verwendet (Graeb et al. 2020a). Zentrales Ziel dieser zusätzlichen Erhebung war es, den initialen Ernährungszustand mithilfe der nutritionDay-Bogen bei Aufnahme zu erfassen. Zusätzlich wurden weitere Ernährungsparameter und Lebensqualität sowie die Handkraft und Körperzusammensetzung per bioelektrischer Impedanzanalyse (BIA) gemessen. Vor der BIA-Messung (Gerät: Seca mBCA 525, Seca Deutschland, Hamburg, Deutschland) mussten die Patient*innen mindestens 10 min liegen. Es wurden Befragungen und Messungen in den ersten 24 h nach Aufnahme und möglichst kurz vor Entlassung durchgeführt, um so die Veränderungen des Ernährungsstatus und der Körperzusammensetzung im Zuge des Klinikaufenthaltes zu ermitteln. Einschlusskriterien waren ein Alter von mindestens 65 Jahren und die Aufnahme in den Projektstationen. Ausschlusskriterien waren lediglich eine fehlende Auskunftsfähigkeit, etwa bei fortgeschrittener Demenz und ein implantierter Herzschrittmacher, weil dann eine Messung der Körperzusammensetzung nicht möglich gewesen wäre. Diese Erhebung fand im September/Oktober in Klinik 1 und im November/Dezember in Klinik 2 statt. Hier wurden also über mehrere Monate Patient*innen direkt nach der Krankenhausaufnahme rekrutiert, während beim nutritionDay alle an einem Stichtag anwesenden Patient*innen um eine Teilnahme gebeten wurden, unabhängig vom ursprünglichen Aufnahmetag.

Bei der jeweiligen Analyse der erhobenen Daten entstand die Idee, explorativ zu untersuchen, inwiefern sich geriatrische Patient*innen, unterteilt in die Gruppen zu Hause und in der Langzeitpflege lebend, hinsichtlich des Ernährungsstatus unterscheiden. Da in beiden Datensätzen der Anteil von in der Langzeitpflege lebenden Patient*innen relativ gering war, wurden die Datensätze zusammengeführt. Es wurde hier also ein kombinierter Datensatz aus nutritionDay-Daten (*n* = 146) und der daran angelehnten separaten Erhebung (*n* = 102) einer Sekundäranalyse unterzogen. Eingeschlossen wurden Patient*innen ab 65 Jahre und älter. Alle Patient*innen waren zum Zeitpunkt der Datenerhebung im Krankenhaus. Es wurde u. a. erfasst, wo diese vor der Einweisung gelebt haben. Aus den Erhebungen wurden, wie beschrieben, die verwendeten nutritionDay-Bogen ausgewertet, sowie, wenn vorhanden, die BIA-Messungen. Rekrutiert wurden Patient*innen einer interdisziplinären internistischen Abteilung (Klinik 1) sowie Patient*innen einer unfallchirurgischen Station mit geriatrischem Schwerpunkt, zwei neurologischen Stationen und einer interdisziplinär internistischen (Komfort‑)Station (Klinik 2). Die Befragung wurde vom Team der Hochschule und von Angestellten der beiden Kliniken gemeinsam durchgeführt.

### Erfassung der Mangelernährung

**Figure 1:**
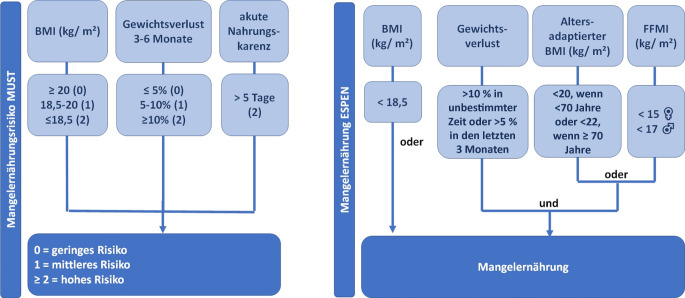

Das Mangelernährungsrisiko wurde anhand des Screeninginstrumentes Malnutrition Universal Screening Tool (MUST) ermittelt. Dieses umfasst die Parameter niedriger BMI, ungewollter Gewichtsverlust in den letzten 3 bis 6 Monaten und Nahrungskarenz über mindestens 5 Tage (Abb. 1). Es wird anhand des ermittelten Scores in geringes, mittleres und hohes Mangelernährungsrisiko unterschieden. Eine manifeste Mangelernährung wurde anhand der Konsensuskriterien der European Society for Clinical Nutrition and Metabolism (ESPEN) identifiziert. Hierzu gehören entweder ein reduzierter BMI oder ein erheblicher Gewichtsverlust in Kombination mit einem altersadaptiert reduzierten BMI oder ein verringerter Fettfreie-Masse-Index (Cederholm et al. 2015). Letzterer, mit FFMI abgekürzt, soll Hinweise auf eine verringerte Muskelmasse geben. Hierfür muss eine Bestimmung der Körperzusammensetzung erfolgen, beispielsweise per BIA. Eine solche Messung liegt für 102 Fälle vor.

### Ethikvotum und Auswertung

Für das gesamte Forschungsprojekt und die darin enthaltenden Erhebungen liegt ein positives ethisches Clearing des Ethikkomitees der Deutschen Gesellschaft für Pflegewissenschaft vor (Antrag-Nr. 17-005). Es wurde stets eine informierte schriftliche Einwilligung der Patient*innen oder ggf. der Betreuer*innen eingeholt. Die Daten wurden vor der Analyse anonymisiert, sodass Rückschlüsse auf die Person nicht mehr möglich sind. Die statistische Analyse wurde mithilfe von IBM Statistics SPSS 26® durchgeführt. Ein *p* < 0,05 wird als signifikant definiert. Es kommen Chi^2^- und Mann-Whitney-U-Test zur Anwendung.

## Ergebnisse

Wesentliche Merkmale der Befragten (*n* = 258) beschreibt Tab. 1. Entsprechend dem Einschlusskriterium ≥ 65 Jahre sind die Patient*innen mit einem Median (Mdn) von 78,0 Jahren (IQR 13) betagt, tendenziell eher weiblich (56,6 %; *n* = 146) und leben zu einem Großteil zu Hause (86,0 %; *n* = 222). Nicht für alle Fälle lassen sich ein Mangelernährungsrisiko und manifeste Mangelernährung einschätzen, da z. T. kein aktuelles Gewicht und BMI vorlagen. Berücksichtigt man nur die Fälle, in denen dies möglich war (*n* = 227), sind 28,8 % (*n* = 64) mit einem hohen und 13,7 % (*n* = 31) mit einem mittleren Mangelernährungsrisiko aufgenommen worden. Ein knappes Viertel weist gemäß den ESPEN-Kriterien eine manifeste Mangelernährung auf (22 %; *n* = 50). Die Patient*innen wurden überwiegend internistisch behandelt (62,8 %; *n* = 162). Die am häufigsten aufgetretenen chronischen Erkrankungen wie Tumorerkrankungen (20,5 %; *n* = 53), Diabetes mellitus (20,2 %; *n* = 62) sowie chronische Niereninsuffizienz (17,8 %; *n* = 46) weisen auf eine alterstypisch ausgeprägte Morbidität hin. Bei 14,7 % (*n* = 38) liegen keine chronische Erkrankungen vor. Im Mittel wird von 4 (IQR 6) Arztkontakten in den vorangegangenen 12 Monaten und 5 (IQR 19) im Krankenhaus verbrachten Nächten berichtet. Betrachtet man ausschließlich die Fälle, die mindestens einmal stationär in der Klinik waren, liegt der Mittelwert bei 14 Nächten (IQR 21,5). Die Angaben zu Klinikaufenthalten und Arztkontakten, in Kombination mit einem ausgeprägten Anteil von Polypharmazie mit mehr als 5 verschiedenen Wirkstoffen am Tag (50,8 %; *n* = 131), sprechen für eine hohe Morbidität. Die geringe Mortalität von 0,8 % (*n* = 2) weist hingegen auf eine weniger schwerwiegende Akuterkrankung als Grund für die Klinikeinweisung hin. Allerdings waren bei der Ermittlung des Outcome nach 4 Wochen noch 9,3 % (*n* = 24) in stationärer Behandlung oder wurden in ein anderes Krankenhaus verlegt, sodass das Outcome in diesen Fällen unklar bleibt. Die mittlere Verweildauer liegt bei 9 Tagen (IQR 11). Bei 7,8 % (*n* = 20) hatte sich im Zuge des Klinikaufenthaltes der Allgemeinzustand so verschlechtert, dass diese nach Entlassung nicht wieder nach Hause konnten, sondern direkt in eine Pflegeeinrichtung einziehen mussten.

**Table 1:** 

Merkmal		*n*		Mittelwert bzw. Anteil
Alter in Jahren	–	258	Mdn (IQR)	78 (13)
Geschlecht	Weiblich	258	% (*n*)	56,6 (146)
Wohnsituation	Zu Hause lebend	258	% (*n*)	86,0 (222)
	Stationäre Pflege		% (*n*)	14,0 (36)
Gewicht in kg	–	236	Mdn (IQR)	69,4 (23,1)
BMI in kg/m^2^	–	227	Mdn (IQR)	24,4 (3,6)
Risiko für eine Mangelernährung (MUST)	Geringes Risiko	227	% (*n*)	58,1 (132)
	Mittleres Risiko		% (*n*)	13,7 (31)
	Hohes Risiko		% (*n*)	28,2 (64)
Manifest mangelernährt	Gemäß ESPEN-Kriterien	227	% (*n*)	22,0 (50)
Aufnahmegrund (ICD-10-Gruppen)	1300 Muskel-Skelett-System und Bindegewebe	258	% (*n*)	15,1 (39)
	0600 Nervensystem		% (*n*)	14,0 (36)
	1000 Atmungssystem		% (*n*)	13,6 (35)
	1900 Verletzungen und Vergiftungen		% (*n*)	10,9 (28)
	0900 Kreislaufsystem		% (*n*)	9,7 (25)
	0200 Neubildungen		% (*n*)	8,1 (21)
	1100 Verdauungssystem		% (*n*)	7,8 (20)
Behandelnde Fachabteilungen	Innere Medizin (interdisziplinär)	258	% (*n*)	62,8 (162)
	Neurologie		% (*n*)	19,8 (51)
	Unfallchirurgie		% (*n*)	17,4 (45)
Chronische Erkrankungen	Tumorerkrankungen	258	% (*n*)	20,5 (53)
	Diabetes mellitus		% (*n*)	20,2 (62)
	Chronische Niereninsuffizienz		% (*n*)	17,8 (46)
	Herzinsuffizienz		% (*n*)	16,7 (43)
	Chronische Lungenerkrankungen		% (*n*)	16,7 (43)
	Zerebrovaskuläre Erkrankung		% (*n*)	16,7 (43)
	Periphere Gefäßerkrankung		% (*n*)	15,9 (41)
Arztkontakte	Letzte 12 Monate	241	Mdn (IQR)	4 (6)
Krankenhausaufenthalte (Anzahl der Nächte)	Letzte 12 Monate	235	Mdn (IQR)	5 (19)
Polypharmazie	> 5 Wirkstoffe/Tag	258	% (*n*)	50,8 (131)
Dauer des Klinikaufenthalts	–	245	Mdn (IQR)	9 (11)
Outcome	Noch im/verlegt in anderes KH	258	% (*n*)	9,3 (24)
	Mortalität (4 Wochen)		% (*n*)	0,8 (2)
	Umzug in stationäre Pflege		% (*n*)	7,8 (20)

*Mdn* Median, *IQR* Interquartilsabstand

### Gruppenvergleich zu Hause vs. stationäre Langzeitpflege

In einem nächsten Schritt werden die Gruppen *zu Hause* und *in einer stationären Pflegeeinrichtung lebend* gegenübergestellt. Die Anteile der verschiedenen chronischen Erkrankungen unterscheiden sich in den meisten Fällen nicht signifikant. Lediglich Tumorerkrankungen sind in der Gruppe der *zu Hause Lebenden* signifikant häufiger anzutreffen (23,0 %; *n* = 51 vs. 5,6 %; *n* = 2; *p* 0,016). Die *in stationären Pflegeeinrichtungen lebenden* Patient*innen (Tab. 2) sind signifikant älter (86 vs. 78 Jahre; *p* < 0,001) und häufiger weiblichen Geschlechts (72,2 % vs. 54,1 %; *p* 0,109), allerdings nicht signifikant. Der längere Klinikaufenthalt (14,5 vs. 8,0 Tage; *p* 0,001), die höhere Anzahl an Medikamenten (9,0 vs. 7,0; *p* 0,002), die geringer ausgeprägte Gehfähigkeit (1,0 vs. 3,0; *p* < 0,001) und der negativer eingeschätzte Gesundheitszustand (3,0 vs. 3,0; mittlerer Rang 125,26 vs. 152,70; *p* 0,034) deuten auf eine höhere Morbidität und körperliche Einschränkungen hin.

**Table 2:** 

	*n* zu Hause/stat. Pflege	Zu Hause	Stat. Pflege	
		Mdn (IQR)	Mdn (IQR)	*p*
Alter in Jahren	222/36	78,0 (12,3)	86,0 (9)	< 0,001
Gewicht in kg	206/30	70,5 (24,4)	62,3 (22)	0,009
BMI in kg/m^2^	199/28	24,6 (7)	23,3 (7)	0,106
Behandlungstage	209/36	8,0 (10)	14,5 (10,5)	0,001
Anzahl der Medikamente	222/36	7,0 (5)	9,0 (5,8)	0,002
Gehfähigkeit^a^	222/35	1,0 (2)	3,0 (0)	< 0,001
Arztkontakte, letzte 12 Monate	214/27	4,0 (6)	5,0 (5)	0,676
Anzahl der Krankenhausnächte in den letzten 12 Monaten	208/27	5,0 (17,8)	10,0 (20)	0,007
Selbsteinschätzung des Gesundheitszustands^b^	222/35	3,0 (2)	3,0 (1)	0,034

^a^ Skala geht von 1 ohne Hilfe gehfähig bis 5 bettlägerig

^b^ Skala von 1 sehr gut bis 5 sehr schlecht

### Gruppenvergleich zum Ernährungsstatus

Der BMI unterscheidet sich nur unwesentlich zwischen den Gruppen *zu Hause* und *in der stationären Langzeitpflege lebend* (23,3 vs. 24,6 kg/m^2^), während das mediane Gewicht mit 62,3 kg signifikant niedriger ist (vs. 70,5 kg; *p* 0,009), was sich durch den höheren Frauenanteil(anteil-) erklären lässt. Beide Gruppen weisen ähnliche Anteile eines Mangelernährungsrisikos auf (Tab. 3). Jedoch sind die *in stationärer Langzeitpflege lebenden* Patient*innen mit 32,1 % etwas häufiger von einem hohen Mangelernährungsrisiko (zu Hause 27,6 %) und mit 35,7 % auch häufiger von einer manifesten Mangelernährung betroffen (zu Hause 20,1 %). Die Unterschiede sind jedoch nicht signifikant.

**Table 3:** 

		Zu Hause		Stat. Pflege		*p*
		%	(*n*)	%	(*n*)	
Mangelernährungsrisiko (MUST)	Gering	58,3	(116)	57,1	(16)	0,825
	Mittel	14,1	(28)	10,7	(3)	
	Hoch	27,6	(55)	32,1	(9)	
Manifest mangelernährt (ESPEN)	–	20,1	(40)	35,7	(10)	0,062

## Diskussion

Die Ergebnisse zeichnen das erwartete Bild einer geriatrischen Population mit relativ hoher Morbidität und hohem Mangelernährungsanteil. Bei Eglseer et al. (2020) liegt der Anteil geriatrischer Patient*innen mit Mangelernährungsrisiko laut MUST-Screening bei vergleichsweise niedrigen 24,3 %, während hier insgesamt 41,9 % ein mittleres bis hohes Mangelernährungsrisiko aufweisen. Betrachtet man dagegen die Ergebnisse des Reviews von Kiesswetter et al. (2016) bewegen sich die Anteile eines Mangelernährungsrisiko (38–65 %) und der Mangelernährung (17–30 %) in einem eher noch höheren Rahmen. In diesem Review werden auch die Gruppen selbstständige Senioren, häuslich gepflegte Senioren und Pflegeheimbewohner verglichen, wobei Letztere den höchsten und selbstständig zu Hause lebende Senioren den geringsten Mangelernährungsanteil aufweisen (Kiesswetter et al. 2016). Dies ist jedoch nicht deckungsgleich mit geriatrischen Patient*innen, da die von Kiesswetter et al. ausgewerteten Studien überwiegend in den jeweiligen Settings, also beispielswiese in stationären Pflegeeinrichtungen, durchgeführt wurden. Die im Review aufgeführten Studien mit geriatrischen Patient*innen im Krankenhaus differenzieren jedoch nicht zwischen zu Hause und in Pflegeeinrichtungen lebenden Personen.

Nun kann angenommen werden, dass in Pflegeeinrichtungen lebende Menschen einen vergleichsweise höheren Pflegebedarf aufweisen. Der Faktor Pflegebedarf stellt einen signifikanten Risikofaktor für die Entwicklung einer Mangelernährung dar (Palm et al. 2012). Somit erscheint es plausibel anzunehmen, dass sich die Subgruppen *zu Hause lebend* und *in der stationären Langzeitpflege lebend* in vielerlei Hinsicht unterscheiden könnten, v. a. aber hinsichtlich Pflegebedürftigkeit und Morbidität und als Folge daraus auch beim Ernährungsstatus. Dass eine steigende Morbidität mit einer zunehmenden Pflegebedürftigkeit assoziiert ist, kann anhand von Routinedaten aufgezeigt werden (van den Bussche et al. 2014).

Im Gruppenvergleich ist in der Gruppe *in der stationären Langzeitpflege lebend* ein höherer Anteil von einer Mangelernährung betroffen. Dieser Unterschied ist jedoch nicht signifikant, möglicherweise aufgrund der zu kleinen Gruppe. Beim Vergleich hinsichtlich eines Mangelernährungsrisikos ist dagegen kein nennenswerter Unterschied auszumachen. Man könnte das Mangelernährungsrisiko gemäß MUST-Kriterien als akute Verschlechterung des Ernährungsstatus betrachten, aus dem sich in der Folge evtl. eine manifeste Mangelernährung entwickelt. Es erscheint unter diesem Gesichtspunkt plausibel, dass sich die hier verglichenen Gruppen geriatrischer Patient*innen, *zu Hause* oder *in einer stationären Pflegeeinrichtung lebend*, nicht unterscheiden, was ein akutes Mangelernährungsrisiko betrifft. Schließlich sind alle Patient*innen dieser Datenanalyse akut erkrankt und haben daher möglicherweise im ähnlichen Maße zuletzt ihre Nahrungszufuhr reduziert, was ja ein wichtiges Kriterium im Instrument MUST darstellt. Wer über 5 Tage nichts gegessen hat kommt damit schon auf 2 Punkte, was dann als hohes Risiko gewertet wird. Ein Nachlassen des Appetits ist als Folge von akuten Erkrankungen wie schweren Infektionen oder Traumata physiologisch (Hartl 2016a) und damit in beiden Gruppen zu erwarten. Anders sieht es aus, was die Grundkonstitution betrifft, also eine bereits manifeste Mangelernährung. Hier erscheint es ebenso plausibel, dass geriatrische Patient*innen, die aufgrund gesundheitlicher Probleme und steigender Pflegebedürftigkeit in der stationären Langzeitpflege leben, auch eher bereits manifest mangelernährt sind und nach erlittenen Gewichtsverlusten dieses nicht wieder aufbauen. Dass Bewohner*innen der Langzeitpflege das einmal verlorene Gewicht nach einer Krankenhausentlassung nicht wieder zurückerlangen, hat sich in einer früheren Datenauswertung des Projektes bereits gezeigt (Graeb et al. 2020).

Im Gruppenvergleich fallen mehrere Unterschiede auf, die ursächlich für einen eher schlechteren Ernährungsstatus in der Gruppe *in stationärer Langzeitpflege lebend* sein könnten. Eine steigende Morbidität und Pflegebedarf sind als direkte oder indirekte Risikofaktoren für ungewollte Gewichtsverluste, reduzierte Nahrungszufuhr, niedrigen BMI und damit eine Mangelernährung zu betrachten (Palm et al. 2012; Graeb et al. 2020; Volkert et al. 2019b). Als Hinweise auf einen generell schlechteren Gesundheitsstatus bei den *in der stationären Langzeitpflege lebenden* Personen können die signifikant höhere Anzahl täglich einzunehmender Medikamente, die größere Anzahl der in den vorangegangenen 12 Monaten im Krankenhaus verbrachten Nächte sowie eine negativere subjektive Einschätzung des Gesundheitszustands betrachtet werden. Dass Hospitalisierungen grundsätzlich das Risiko für eine Mangelernährung erhöhen, konnte in anderen Studien bereits aufgezeigt werden (Streicher et al. 2018) und war auch Ausgangspunkt dieses Forschungsprojektes. Hier sollte aber angemerkt werden, dass es überwiegend unklar bleibt, ob eine Mangelernährung zu einem Klinikaufenthalt führt, oder der Klinikaufenthalt bzw. die Akuterkrankung zu einer Verschlechterung des Ernährungsstatus. Die im Projekt durchgeführten Erhebungen deuten aber an, dass beides zutreffend ist (Graeb, Wolke und Reiber 2020a; Graeb et al. 2020).

### Limitationen und weiterer Forschungsbedarf

Die durchgeführte Datenanalyse weist ein paar wesentliche Limitation auf. Zum einen ist unklar, inwiefern das unterschiedliche Vorgehen bei den Erhebungen, also der Stichtagserhebung im Rahmen des nutritionDay vs. einer Erhebung über einen längeren Zeitraum, die Ergebnisse beeinflusst haben könnten. In allen Erhebungen war es außerdem schwierig, schwer Pflegebedürftige zu rekrutieren, da diese oftmals nicht ausreichend auskunftsfähig waren. Auch eine fehlende Einwilligungsfähigkeit ist hierbei problematisch. In solchen Fällen wurden zwar die Betreuer*innen angesprochen, was im Vorfeld nicht immer rechtzeitig gelang, weswegen speziell Patient*innen mit fortgeschrittener Demenz in der Stichprobe völlig unterrepräsentiert sind. Allerdings ist eine Demenz in der Literatur ein wesentlicher Risikofaktor für die Entwicklung einer Mangelernährung (Reuther et al. 2013). Auch lässt schon die niedrige Mortalität von 0,8 % darauf schließen, dass die eingeschlossenen Patient*innen nicht schwer akut erkrankt sind, was die Repräsentativität ebenfalls etwas einschränkt. Zum Vergleich, in einer groß angelegten Analyse von nutritionDay-Daten liegt die Gesamtmortalität im Krankenhaus bei 3,6 % (Cereda et al. 2017). Eine weitere Limitation betrifft die nichtvollständigen Daten. So fehlen teilweise Gewichtsdaten (nutritionDay-Datensatz) und Angaben zu Fragen, die von den Betroffenen nicht beantwortet werden konnten oder auf die die Befragten nicht antworten wollten. Ein Nachteil von Sekundäranalysen besteht nun darin, dass solche Lücken im Nachhinein nicht mehr ausgefüllt werden können. Dieses Vorgehen macht es daher auch unmöglich, neuere Kriterien zu berücksichtigen. So wurden erst 2019 die Kriterien der Global Leadership Initiative on Malnutrition (GLIM) zur Bestimmung einer Mangelernährung von mehreren internationalen Fachgesellschaften gemeinsam konsentiert und der Fachöffentlichkeit vorgestellt (Cederholm et al. 2019). Aufgrund der dafür erforderlichen Datenbasis konnten diese aber hier nicht zur Anwendung kommen.

Die vorliegenden Daten ließen die Anwendung eines Morbiditätsindex zur besseren Quantifizierung einer Morbidität nicht zu, jedoch deuten die eher häufiger vorkommenden chronischen Erkrankungen in der Gruppe stationäre Pflege auch auf eine ausgeprägtere Morbidität hin. Ein höherer Pflegebedarf lässt sich zwar anhand der signifikant geringeren Gehfähigkeit vermuten. Allerdings konnten ggf. vorhandene Pflegegradeinstufungen ebenfalls nicht miterfasst werden.

Auch aufgrund dieser Limitationen wären weitere Studien wünschenswert. Vor allem die inzwischen rasant angewachsene Datenbank des nutritionDay-Projektes würde hier zahlreiche Möglichkeiten bieten, speziell geriatrische Patient*innen näher unter die Lupe zu nehmen und dabei, wie hier, zwischen der Herkunft aus den einzelnen Settings zu unterscheiden. Auch eine Analyse speziell der Fälle, die nach Entlassung aus dem Krankenhaus in eine stationäre Pflege umziehen mussten, wäre in jedem Fall interessant.

## Empfehlungen für die Praxis

Beide Gruppen weisen ähnlich hohe Anteile eines akuten Mangelernährungsrisikos auf. Das bedeutet, dass in der klinischen Praxis generell ein stärkerer Fokus auf diese Problematik gelegt werden sollte, indem dieses Risiko mithilfe eines Screenings systematisch erfasst wird. Dass dies bislang eher nicht geschieht, wurde eingangs bereits aufgezeigt. Ferner deutet sich bei der manifesten Mangelernährung ein höherer Anteil bei den *in der stationären Langzeitpflege lebenden* Patient*innen an, auch wenn dieser Unterschied nicht signifikant ist. Ein solcher Unterschied würde aber nicht bedeuten, dass die Unterbringung in stationären Pflegeeinrichtungen ursächlich für einen sich verschlechternden Ernährungsstatus wären. Es lässt sich so lediglich eine erheblich höhere Vulnerabilität der dort lebenden Senior*innen für eine Mangelernährung ableiten, mit dem impliziten Auftrag, den Ernährungsstatus im Blick zu behalten, also regelmäßig zu erfassen und ggf. zu intervenieren. Dass dies aber nicht immer zuverlässig gelingt, darauf deuten Analysen von Routinedaten in der stationären Altenpflege hin (Graeb et al. 2020b).

Allerdings sind bereits auch 20,1 % der *zu Hause lebenden* geriatrischen Patient*innen von einer Mangelernährung betroffen. Das Problem betrifft damit grundsätzlich häufig geriatrische Patient*innen, in jedem Versorgungskontext. Aktuelle Arbeiten weisen darauf hin, dass schwere Verläufe bei COVID-19 mit Mangelernährung assoziiert sind (Bedock et al. 2020; Pironi et al. 2020). Es ist außerdem bekannt, dass ungewollte Gewichtsverluste und andere Anzeichen einer Mangelernährung das Mortalitätsrisiko im Krankenhaus allgemein erhöhen (Barazzoni et al. 2020; Hiesmayr et al. 2019), was die Problematik der Mangelernährung noch einmal verdeutlicht. Gleichzeitig können gezielte Interventionen bei mangelernährten Patient*innen die Mortalität wiederum signifikant senken (Schuetz et al. 2019). Für diese Interventionen ist jedoch das Screening zum Erkennen einer Mangelernährung bzw. eines Mangelernährungsrisikos die notwendige Grundlage.

Für die stationäre Langzeitpflege und auch den ambulanten Bereich ist es somit v. a. wichtig, möglichst frühzeitig Hinweise auf Mangelernährungsrisiken zu erkennen, um dem entgegenzuwirken, also präventiv tätig werden zu können, um so schwerwiegende Folgen für die Betroffenen zu vermeiden oder zumindest abzumildern.
